# Mechanical Behavior of Shape Memory Alloy Fibers Embedded in Engineered Cementitious Composite Matrix under Cyclic Pullout Loads

**DOI:** 10.3390/ma15134531

**Published:** 2022-06-27

**Authors:** Zhao Yang, Yalong Du, Yujia Liang, Xiaolong Ke

**Affiliations:** 1 School of Urban Construction, Wuhan University of Science and Technology, Wuhan 430065, China; adyldyx@163.com (Y.D.); andrea_liang663@126.com (Y.L.); m15623266213@163.com (X.K.); 2 Institute of High Performance Engineering Structure, Wuhan University of Science and Technology, Wuhan 430065, China

**Keywords:** SMA, ECC, superelastic, mechanical behavior, self-centering

## Abstract

The incorporation of superelastic shape memory alloy (SMA) fibers into engineered cementitious composite (ECC) materials can provide high seismic energy dissipation and deformation self-centering capabilities for ECC materials. Whether the SMA fibers can be sufficiently bonded or anchored in the ECC matrix and whether the mechanical properties of the SMA fibers in the ECC matrix can be effectively utilized are the key scientific issues that urgently need to be studied. In order to study the mechanical behavior of SMA fiber embedded in ECC matrix, four groups of semi-dog-bone pullout specimens were fabricated, and the cyclic pullout tests were conducted in this paper. The pullout stress, displacement, and self-centering capability were analyzed, and different influencing factors were discussed. The results show that the knotted ends can provide sufficient anchorage force for SMA fibers, and the maximum pullout stress of SMA fiber can reach 1100 MPa, thus the superelasticity can be effectively stimulated. The SMA fibers show excellent self-centering capability in the test. The minimum residual deformation in the test is only 0.29 mm, and the maximum self-centering ratio can reach 0.93. Increasing bond length can increase the ultimate strain of SMA fibers with knotted ends, but reduce the maximum pullout stress. Increasing fiber diameter can increase both the ultimate strain and the maximum stress of knotted end SMA fibers. While neither bond length nor fiber diameter has significant effect on the self-centering ratio. This paper provides a theoretical basis for further study of the combination of SMA fibers and ECC materials.

## 1. Introduction

Concrete is the most widely used building material today. However, traditional concrete materials have low tensile strength, poor ductility, and are prone to cracking, which can easily cause the corrosion of internal steel bars, thereby reducing the durability of structures and components. In order to meet the requirements of modern construction and overcome the defects of traditional concrete, scholars around the world have carried out a great deal of research work to improve traditional concrete or cement-based materials in order to form new building materials with good tensile strength, ductility and durability [1,2,3].

Engineered cementitious composite (ECC) is a kind of super-ductile composite. ECC has high toughness and fracture resistance due to its strain-hardening property. Its ultimate tensile strain can reach 3–5%, and its strain capacity is 300–500 times that of ordinary concrete. During the tensile process of ECC, a series of micro-cracks with a width less than 100 μm appeared, and the crack spacing was less than 3 mm, showing a multi-cracked strain hardening state [4,5,6,7]. ECC can effectively improve the seismic performance of engineering structures. Yuan et al. [8] studied the flexural performance of the hybrid reinforced beam through the four-point bending test, and found that replacing concrete with ECC in the beam could improve its bearing capacity and ductility. When ECC was poured in the tensile area, the average crack width and crack spacing of the beam would be reduced, which could fully protect the longitudinal reinforcement from corrosion. Xu et al. [9] used ECC to replace concrete in the plastic hinge region of an RC-ECC column, and conducted a cyclic quasi-static test to study its seismic performance. The results show that RC-ECC columns can provide better energy dissipation capacity and higher ultimate bearing capacity than concrete columns. Dong et al. [10] studied the seismic performance of the joint through the cyclic loading experiment, and found that, compared with the concrete specimen, the specimen using ECC in the joint area had higher bearing capacity, ductility and energy dissipation capacity. However, the residual deformation of ECC components after loading is significant, and their self-centering performance is poor.

Shape memory alloy (SMA) is one of the most widely studied and applied intelligent materials at present. Because of its excellent shape memory effect, superelasticity, high damping property, good biocompatibility and corrosion resistance, SMA is widely used in aerospace, mechanical and military industries, biomedicine and other fields [11,12,13,14]. Superelastic SMA has flag-shaped hysteretic energy dissipation characteristics and self-recovery ability after unloading. Therefore, it has been widely used in self-centering seismic structures in recent years. Jia et al. [15] studied the seismic performance of SMA brace specimens through a cyclic loading test. The test results show that the SMA brace has good energy dissipation capacity, bearing capacity and self-centering ability. Abraik et al. [16] studied the influence of different design parameters on the lateral shear resistance of concrete shear wall. The test results show that the use of SMA-FRP hybrid reinforced concrete not only solves the problem of wall durability, but also significantly improves the seismic performance.

Some scholars used superelastic SMA to strengthen ECC materials, and achieved obvious results in improving the seismic energy dissipation capacity and deformation self-centering capacity of ECC [17]. However, the current main application form of SMA materials is continuous bars or rods, which poses some problems due to its high price, difficulty in processing and formation, the need to make special connectors to connect with steel bars, and because it can be prone to the development of weak sections. Using SMA fibers can solve these problems. In addition, by incorporating SMA fibers into ECC materials, the uniform distribution of SMA fibers can provide recovering force for ECC materials in a wider range, which is more suitable for the wide-range cracking characteristics of ECC [18]. The study conducted by the author has showed that the incorporation of superelastic SMA fibers into ECC materials could provide high seismic energy dissipation and deformation self-recovery ability for ECC materials [19].

However, due to the smooth surface of SMA fibers, the bonding strength between SMA fibers and the ECC matrix is very low. When subjected to stress, the SMA fibers and the ECC matrix are prone to premature debonding and failure, thus the SMA fibers cannot provide superelasticity for ECC. Therefore, ensuring that the SMA fibers can obtain sufficient bonds or anchorages in the ECC matrix is the basis for taking full advantage of the superelasticity of SMA. However, there are no studies on the bonding or anchoring of SMA fibers to ECC matrix in the current literature, and there is no related study on the mechanical behavior of SMA fibers embedded in ECC matrix.

Therefore, this paper adopts three different SMA fiber end shapes, hoping to provide sufficient anchorage force for SMA fiber embedded in ECC matrix by setting the ends. On this basis, the cyclic pullout tests were carried out. By analyzing the pullout stress of SMA fibers, it was investigated whether the superelasticity of SMA fibers could be effectively exerted. By comparing the pullout stress of SMA fibers with different SMA fiber end shapes, different fiber diameters and different bonding lengths, the main influencing factors were studied. By analyzing the self-centering ratio, the self-centering ability of different specimens was studied as well. Through the above studies, the mechanical behavior of the SMA fibers embedded in the ECC matrix can be clarified, which provided a theoretical basis for further research.

## 2. Experiment Design

### 2.1. Materials

The main materials for formulating ECC in this study include type I Portland cement, type F and grade I fly ash, silica sand, water, water reducing agent and PVA short fibers. Among them, the measured compressive strength of cement standard test block after 28 days of curing is 42.5 MPa, the fineness of fly ash is 43 μm, the particle diameter of silica sand is between 0.075 mm and 0.15 mm, and the water reducing agent is polycarboxylate superplasticizer (PS). The parameters of PVA fibers are shown in Table 1, where the values were obtained from the material supplier. The mixture weight proportion of ECC material is shown in Table 2.

**Figure 1:**
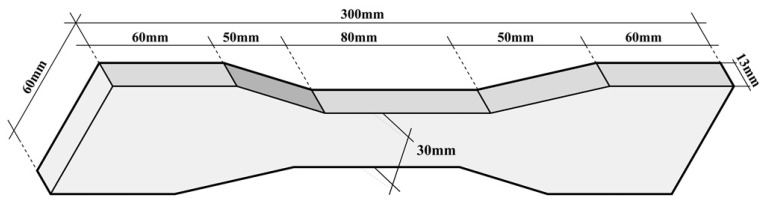
Specific size of tensile specimen.

According to Chinese Standard JC/T 2461-2018: *Standard test method for the mechanical properties of ductile fiber reinforced cementitious composites*, the dog-bone shaped specimens were made according to the mix proportion shown in Table 2 for tensile test through a test machine WE-1000A to obtain the tensile properties of ECC material. The specific size of the ECC dog-bone shaped specimen is shown in Figure 1. The curing time for these ECC specimens was 28 days, the curing temperature and relative humidity were 20 ± 1 °C and 50 ± 5% respectively.

The tensile test was carried out though an electro-hydraulic servo universal testing machine. The loading was controlled by displacement with a 0.2 mm/min loading rate and was stopped after the main crack appeared.

**Figure 2:**
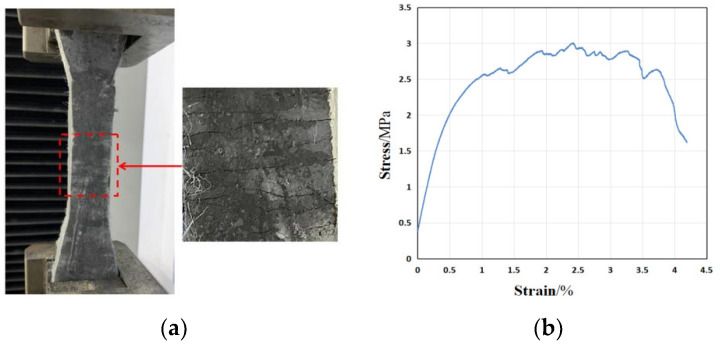
Test results of the ECC specimen: (**a**) Cracks; (**b**) Stress–strain curve.

During the tensile process, multiple small cracks appeared in the middle tensile area of the dog-bone shaped specimen as shown in Figure 2a. The tensile stress–strain curve of ECC specimen is shown in Figure 2b. It can be seen from Figure 2b that the ECC specimen showed good ductility in the tensile process, and the ultimate strain (corresponding strain when the stress drops to 85% of the peak stress) reached 3.12%. In addition, obvious fluctuations can be seen in the stress–strain curve, indicating that new cracks continued to appear in the specimen and PVA fibers played the bridging role, providing the strain-hardening characteristics for ECC material. The tensile strength of the ECC specimen reached 3.0 MPa.

The diameters of the SMA wires used in the test were 1.0 mm, 1.2 mm and 1.5 mm, respectively. The main material composition of the SMA is 55.86% Ni and 44.14% Ti. The start temperature and the end temperature of austenite phase transformation is −34.60 °C and −18.19 °C, respectively. Therefore, the Ni-Ti SMA will be in the austenitic state at room temperature.

**Figure 3:**
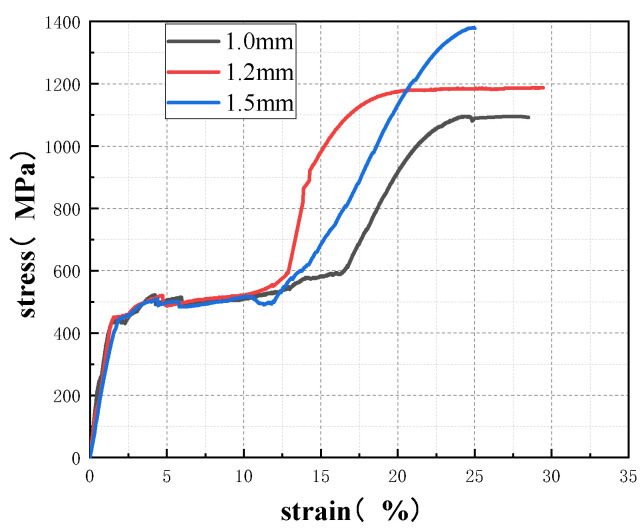
Direct tensile stress–strain curves of SMA wires with different diameter.

**Figure 4:**
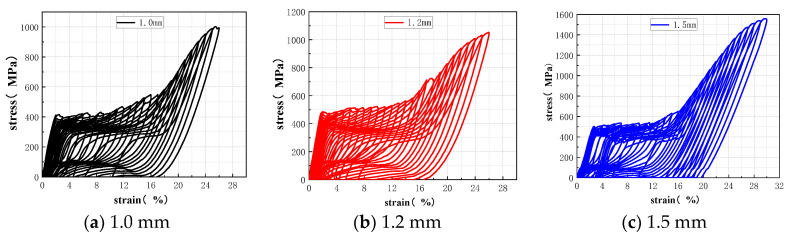
Cyclic tensile stress–strain curves of SMA wires with different diameter: (**a**) Diameter is 1.0 mm; (**b**) Diameter is 1.2 mm; (**c**) Diameter is 1.5 mm.

In order to obtain the tensile properties of the SMA wires, the uniaxial direct tensile test and the cyclic tensile test were carried out, respectively. The uniaxial tensile stress–strain curves of SMA wires with different diameter are shown in Figure 3, and the mechanical properties are shown in Table 3, the cyclic tensile stress–strain curves of SMA wires with different diameter are shown in Figure 4.

### 2.2. Specimen Design and Fabrication

**Figure 5:**
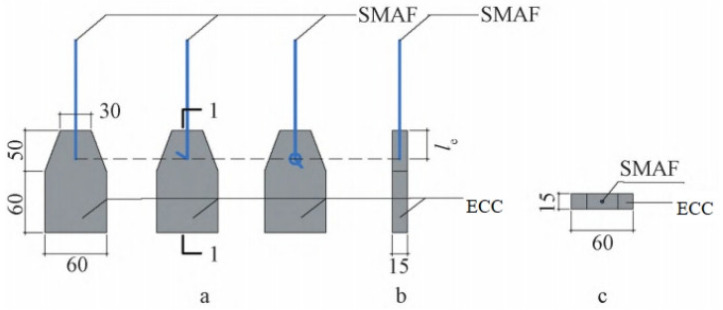
Specific size of the semi-dog-bone drawing specimen: (**a**) Front view (from left to right are straight specimens, hooked specimens, and knotted specimens); (**b**) 1-1 section (l_e_ is the bonding length); (**c**) Top view. (Note: The unit for the numbers in the figure is mm.)

**Figure 6:**
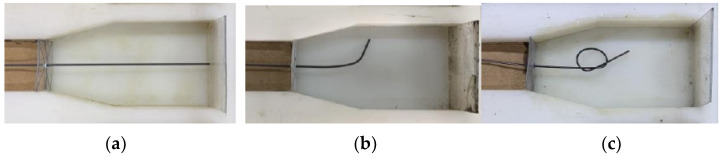
Placement of SMA fibers with different end shape: (**a**) Straight end; (**b**) Hook end; (**c**) Knotted end.

In order to study the bonding mechanical behavior of SMA fiber and ECC matrix, the cyclic drawing tests were carried out. The SMA fiber diameter, end shape and bonding length were selected as the main influencing factors for the design and manufacture of semi-dog-bone drawing specimens. The diameters of SMA fibers were selected as 1.0 mm, 1.2 mm and 1.5 mm. The end shapes were set to be straight, hooked and knotted. The bond lengths were set to 30 mm, 40 mm and 50 mm. The calculation of bond length between SMA fiber and ECC matrix does not include the length of hook and knot. Table 4 shows the specimen grouping table. Figure 5 shows the specific size of the semi-dog-bone drawing specimen. Figure 6 shows the placement of SMA fibers with different end shape during the test. The ECC mix ratio, fabricated and curing process are the same as that mentioned in Section 2.1.

### 2.3. Test Device and Test Method

**Figure 7:**
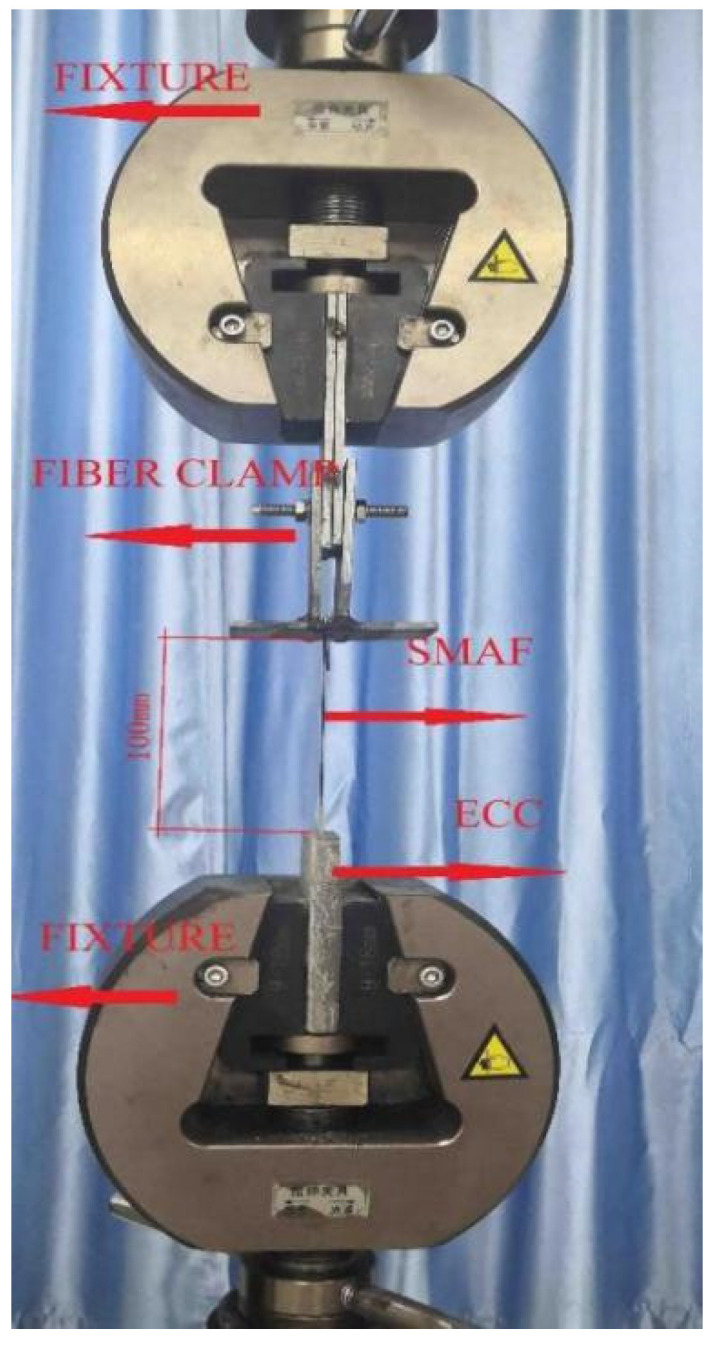
The device of the cyclic drawing test.

A universal testing machine was used for the cyclic test, and the test device was shown in Figure 7. The ECC part of the specimen was placed in the fixture at the bottom of the universal testing machine, and the free end of the SMA fiber is fixed by a fiber clamp which was placed in the fixture at the top of the testing machine. The length of the free tensile section was 100 mm. The built-in sensor of the testing machine was used to record the load and the pulling displacement during the test. The whole test process was controlled and the test data was synchronously collected by the computer.

The loading was controlled by displacement with a 0.5 mm/min loading rate. The cyclic loading of each stage is 1 mm. The loading will be stopped when meeting the following condition: (1) SMA fiber strain reached more than 30%; (2) SMA fiber fractured; (3) The test instrument showed that the drawing load was negative.

## 3. Test Results

### 3.1. Pullout Stress–Displacement Curve

**Figure 8:**
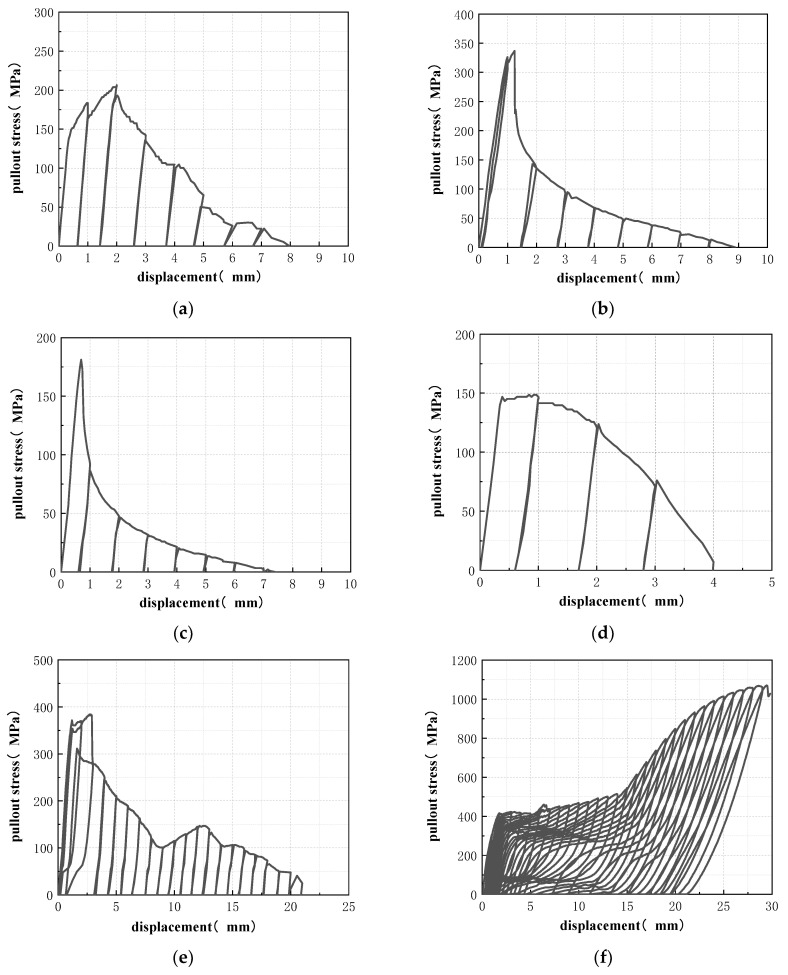

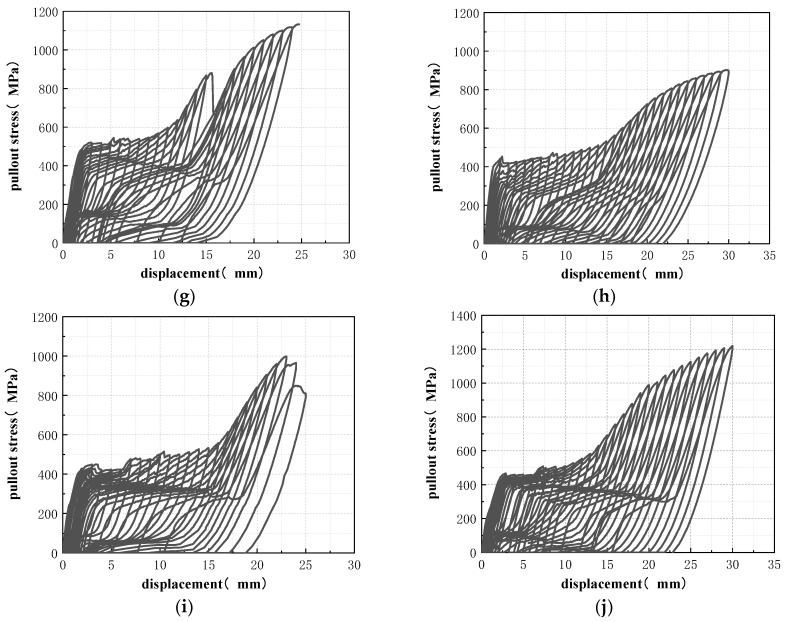
Cyclic pullout stress–displacement curve of the specimens: (**a**) st-1.0; (**b**) st-1.2; (**c**) st-1.5; (**d**) s-1.2-40; (**e**) h-1.2-40; (**f**) k-1.2-40; (**g**) k-1.2-30; (**h**) k-1.2-50; (**i**) k-1.0-40; (**j**) k-1.5-40.

By analyzing the pull-out stress of SMA fibers, it is possible to analyze what level of SMA fiber stress can reach before the anchoring failure of the SMA fiber and the ECC matrix, and whether the SMA fiber can exceed the phase transition stress to effectively stimulate superelasticity. The pullout stress–displacement curves of specimens in the cyclic drawing tests are shown in Figure 8. *σ_f,max_* is the maximum pullout stress of the SMA fiber and its expression is shown in Equation (1).

(1) $$\sigma_{f,max}=\frac{F_{max}}{A_{f}}=\frac{F_{max}}{\pi \cdot \frac{d_{f}^{2}}{4}}$$

where, *F_max_* is maximum drawing load; *A_f_* is the cross-sectional area of the SMA fiber; *d_f_* is diameter of the SMA fiber.

From Figure 8, it can be seen that the pullout stress–displacement curves of the specimens with straight ends SMA fiber (st-1.0, st-1.2, st-1.5, s-1.2-40) and hooked ends SMA fiber (h-1.2-40) did not show phase change stress platforms, and the residual deformation after unloading was not reduced. The SMA fibers were pulled out obviously when the semi-dog-bone specimens were broken, and the ECC matrix was not damaged. While the pullout stress-displacement curves of specimens with knotted ends SMA fiber (k-1.2-40, k-1.2-30, k-1.2-50, k-1.0-40, k-1.5-40) showed obvious phase change stress platform, and the residual deformation decreased significantly after unloading. Among them, the minimum residual deformation of the specimen k-1.0-40 in the phase transformation stage is only 0.29 mm, showing excellent deformation recovery ability. When the specimens were damaged, the SMA fibers were broken, and the ECC matrix was not damaged. The results show that the straight and hook ends cannot provide enough anchorage for SMA fiber, which leads to the premature pull-out of SMA fiber from ECC matrix. The stress of SMA fiber cannot reach the phase transformation stress, and cannot produce superelasticity. While the knotted end can provide enough anchoring force for SMA fiber, and the stress of SMA fiber can continue to increase to the phase transition stress. Therefore, SMA fiber can produce superelasticity, which can effectively reduce the residual deformation of the test specimens.

### 3.2. Analysis on the Influence Factors of Stress and Strain

#### 3.2.1. Diameter of the SMA Fiber with Straight Ends

The first group of specimens includes specimen st-1.0, st-1.2 and st-1.5. The specimens all adopted straight SMA fiber ends, but the fiber diameters were 1.0 mm, 1.2 mm and 1.5 mm, respectively. It can be seen from Figure 8a–c that with the increase of loading displacement, the pullout stress of SMA fiber of the three specimens quickly reached the maximum stress, and the corresponding loading displacement was less than 2 mm. Among these three specimens, the specimen with 1.2 mm fiber diameter (st-1.2) had the highest peak stress and ultimate displacement that is nearly 350 MPa and 9 mm respectively. This phenomenon shows that for this group of specimens, the bonding force between the SMA fibers and the ECC matrix is mainly derived from the adhesion and surface friction. When the diameter of the SMA fiber is larger, the bonding area between the SMA fiber and the ECC matrix is larger, and the bonding force provided by the bonding interface is larger accordingly. However, the larger the diameter of the SMA fiber, the larger the cross-sectional area of the fiber. Therefore, the pullout stress of the SMA fiber does not necessarily increase with the increase of the fiber diameter. The results of this test show that when the diameter of the SMA fiber increases to 1.2 mm, continuing to increase the diameter of the SMA fiber will increase the negative influence of the SMA fiber on the original structure of the ECC matrix, thereby reducing the strength of the ECC matrix around the SMA fiber to a certain extent, resulting in the interface bonding strength decreases, and lead to a smaller increase in the bonding force than the increase in the bonding area, so the pullout stress of the SMA fiber becomes smaller.

With the increase of loading displacement, the pullout stress of SMA fiber decreased rapidly. There was no phase change stress platform appeared in the stress-displacement curves. The residual deformation of the specimens did not decrease after unloading. When the specimens were damaged, the SMA fibers were pulled out, and there was no obvious damage to the ECC matrix. The above phenomenon shows that the straight end cannot provide enough bonding and anchoring force for SMA fibers. When loading, the interface between smooth SMA fiber and ECC matrix prematurely fails, resulting in the pull-out of SMA fiber and the failure of semi-dog-bone drawing specimens. Although the fiber diameter has a significant effect on the fiber stress, the fiber stress of all specimens with straight fiber ends is still far lower than the phase change stress. Therefore, SMA fiber cannot produce superelasticity.

#### 3.2.2. End Shape of the SMA Fiber

The second group of specimens includes specimens s-1.2-40, h-1.2-40 and k-1.2-40. The specimens adopted straight ends, hooked ends and knotted ends respectively, but the same fiber diameter of 1.2 mm and the same bond length of 40 mm.

From Figure 8d, it can be seen that the SMA fiber stress of the straight end SMA fiber specimen (s-1.2-40) quickly reached the maximum value, and then the fiber stress decreased rapidly. There was no phase transformation stress platform, and the residual strain did not decrease, the SMA fiber could not produce superelasticity. The test results of this specimen are very similar to those of the first group, but because the bond length of this specimen is smaller, its maximum stress and ultimate displacement are also smaller than those of the first group. When compared with the specimen st-1.2 (Figure 8b), the only difference between the two specimens is the bonding length. The maximum pullout stress of the SMA fiber in the specimen with a bonding length of 40 mm (s-1.2-40) is only about 43% of the specimen with a bonding length of 110 mm (st-1.2), and the ultimate displacement is only about 44% of that specimen. It shows that, for the straight SMA fibers, whose bonding force is mainly composed of adhesive force and friction force, the influence of the bond length on the bond force is very significant.

Figure 8e is for the hooked end SMA fiber specimen (h-1.2-40), the stress of SMA fiber reached the maximum value in the second loading cycle, and the stress value in the third loading cycle had a significant sudden decrease compared with that in the previous cycle. Then, the stress of SMA fiber continued to decrease, and there was no phase change stress platform. The residual strain did not decrease significantly, and there was no obvious superelasticity generated. However, compared with the straight end specimen (s-1.2-40), the maximum fiber stress of the hooked end specimen (h-1.2-40) was significantly increased, close to 400 MPa. Then, after the stress concentration occurred at the hooked end, sufficient anchoring force could not be provided, resulting in the pullout of SMA fiber when the failure occurred.

Figure 8f is for the knotted end SMA fiber specimen (k-1.2-40), the stress-displacement curve has obvious flag shape characteristics. When the stress reached about 400 MPa, the phase change platform started. When the stress reached 450 MPa, the phase change platform ended. Then, the slope of the curve increased significantly. Finally, the fiber was broken when the displacement reached 29 mm. The maximum pullout stress of the SMA fiber in this specimen was more than 1000 MPa, that was about 7 times that of the straight end fiber and 2.7 times that of the hooked end fiber. Therefore, the knotted end can provide sufficient anchoring force for the SMA fiber, the SMA fiber can reach the phase transformation platform stress and enter the martensite hardening state, thus the superelasticity can be generated effectively.

#### 3.2.3. Bond Length of the SMA Fiber with Knotted Ends

The third group of specimens includes specimens k-1.2-30, k-1.2-40 and k-1.2-50. The specimens all adopted knotted SMA fiber ends, with the same fiber diameter of 1.2 mm, but different bond length that is 30 mm, 40 mm and 50 mm, respectively. It can be seen from Figure 8f–h that the curves of these three specimens showed obvious flag-shaped characteristics and the phase transformation platform. Therefore, all of the three bond lengths can provide sufficient anchorage force for SMA fibers. The maximum pullout stress of SMA fiber in the specimen with 30 mm bond length (k-1.2-30) could reach 1100 MPa, but the ultimate displacement is less than 25 mm. The maximum pullout stresses of the other two specimens were lower than k-1.2-30, noted as 1080 MPa for the specimen with 40 mm bond length (k-1.2-40) and 900 MPa for the specimen with 50 mm bond length (k-1.2-50). However, the ultimate displacements of the last two specimens were larger, both noted as nearly 30 mm. The result shows that increasing the bond length can improve the ultimate strain of the SMA fiber, but will reduce the maximum pullout stress. The main reason for this result is that the loading control method in this test is displacement control. When the stress of the SMA fiber exceeds the stress range of the phase transition platform, the fiber has produced a large strain. When the strain level is the same, the longer the bond length of the SMA fiber, the greater the deformation, and the smaller the load required to stretch the SMA fiber to the set displacement value. Therefore, under the same loading displacement, the longer the bond length of the fiber, the smaller the corresponding SMA fiber pullout stress. The specimen k-1.2-30 has the smallest bond length, so the SMA fiber deformation is the smallest. When the load reaches 25 mm, the maximum pullout stress is reached, and the fiber is broken. However, the specimens k-1.2-40 and k-1.2-50 have a larger bond length, so the total fiber deformation is larger, and the fibers have not been broken when the loading displacement reaches 30 mm, indicating that the fiber pullout stress at this time does not reach the maximum value. When the loading displacement is 30 mm, the tensile stress of the SMA fiber of the specimen with a larger bonding length (k-1.2-50) is smaller.

#### 3.2.4. Diameter of the SMA Fiber with Knotted Ends

The fourth group of specimens includes specimen k-1.0-40, k-1.2-40 and k-1.5-40. The specimens all adopted knotted SMA fiber ends, with the same bond length of 40 mm but different fiber diameters, which are 1.0 mm, 1.2 mm and 1.5 mm, respectively. From Figure 8f,i,j, both the flag-shaped characteristics and phase transformation platform can be seen significantly in the curves of these three specimens. Therefore, all of the three fiber diameters can provide sufficient anchorage force for SMA fibers. The maximum pullout stress of the SMA fiber in the knotted specimen with diameter of 1.0 mm (k-1.0-40) is 1000 MPa. While the maximum pullout stress of SMA fiber in the specimen with diameter of 1.2 mm and 1.5 mm was 1090 MPa and 1200 MPa, respectively. In addition, the ultimate displacement of the specimens with larger fiber diameter was also higher than the specimen with smaller fiber diameter. The result shows that increasing the fiber diameter can increase both maximum pullout stress and the ultimate strain of the SMA fiber.

### 3.3. Self-Centering Ratio

In this study, the self-centering ratio was used to evaluate the self-centering performance of the SMA fibers placed in the ECC matrix. The self-centering ratio *u* is calculated as Equation (2).

(2) $$u=\frac{\Delta_{1}-\Delta_{0}}{\Delta_{1}},$$

where $\Delta_{1}$ means the maximum loading displacement in each cycle, $\Delta_{0}$ means the residual displacement after unloading in each cycle.

**Figure 9:**
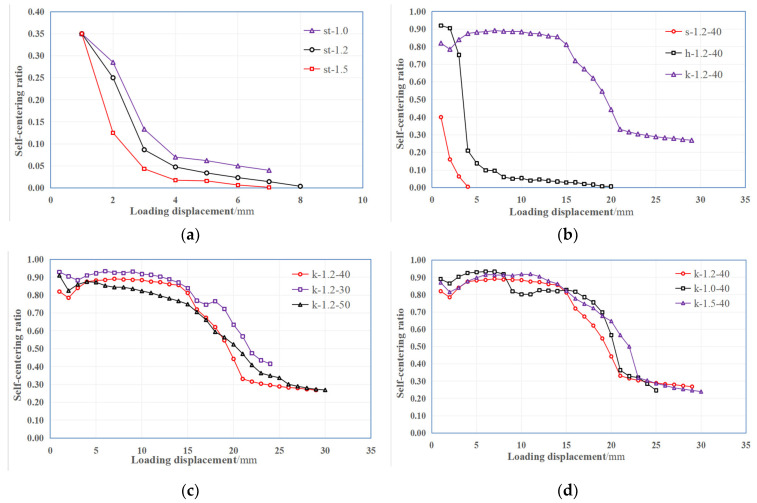
Self-centering ratio curves of the four specimen groups: (**a**) Group 1; (**b**) Group 2; (**c**) Group 3; (**d**) Group 4.

The self-centering ratio curves of the four specimen groups are shown in Figure 9. As shown in Figure 9a, the self-centering ratios of the specimens in group 1 are very low, the highest self-centering ratio of 0.35 appeared in the first cycle when the bonding had not been destroyed. Then with the load increasing, the bonding was destroyed and the self-centering ratio decreased quickly. This result indicates that SMA fibers with straight ends cannot produce self-centering capability in ECC matrix.

As shown in Figure 9b, the specimens with straight end (s-1.2-40) and hooked end (h-1.2-40) SMA fibers had no self-centering ability with the loading displacement increasing, while the specimen with knotted end SMA fiber (k-1.2-40) showed excellent self-centering capability. Before the loading displacement reached 15 mm, the self-centering ratio kept being above 0.8, especially when the loading displacement was in the range of 6 mm to 9 mm, the self-centering ratio reached a maximum value of 0.89. When the loading displacement reached 15 mm, the self-centering ratio of the specimen decreased continuously. It shows that when the fiber stress is in the phase transformation plateau stress, the SMA fiber can obtain the best self-centering ability. When the fiber stress further increased and the SMA was in the martensitic hardening state, the self-centering ability of the SMA fiber began to decrease. This is also reflected in Figure 9c,d.

As shown in Figure 9c, during the phase transformation stage with the loading displacement increased from 3 mm to 15 mm, the specimen with the smallest bond length (k-1.2-30) had the highest self-centering ratio of 0.93, and the maximum ratio of k-1.2-40 and k-1.2-50 is 0.89 and 0.88. This result shows that the bond length has some influence on the self-centering ability of SMA fibers. The specimen with a smaller bond length obtains the maximum self-centering ability, but the difference in the maximum self-centering ratio of the three specimens is within 6%.

As shown in Figure 9d, the effect of fiber diameter on self-centering ability did not show obvious regularity. The specimen with a fiber diameter of 1.0 mm (k-1.0-40) obtained the highest self-centering ratio of 0.93. The self-centering ratio of specimen with fiber diameter of 1.5 mm (k-1.5-40) and 1.2 mm (k-1.2-40) could reach 0.92 and 0.89, respectively. The difference of the self-centering ratio of these three specimens is also very small. The above results indicated that the fiber diameter had no significant effect on the self-centering ability of SMA fibers.

## 4. Conclusions

In this paper, the mechanical behavior of SMA fibers in the ECC matrix under cyclic pullout loads were studied. Four groups of semi-dog-bone pullout specimens were fabricated and cyclic pullout tests were conducted to study the fiber stress, displacement, self-centering capability and influencing factors. The main conclusions are as follows:

1. When the SMA fiber can obtain sufficient anchoring force in the ECC matrix, the superelasticity of SMA can be effectively generated with the increase of fiber stress, and the SMA fiber shows significant flag-shaped energy dissipation characteristics and deformation self-centering ability. The minimum residual deformation of the test specimens during the phase transformation stage is only 0.29 mm.
2. The knotted end can provide sufficient anchoring force for SMA fibers. The maximum pullout stress of SMA fibers in the ECC matrix can reach 1100 MPa, and the maximum pullout displacement can reach 30 mm. Therefore, the superelasticity of SMA fibers can be effectively stimulated.
3. Increasing the bond length of straight end SMA fiber can improve both the maximum pullout stress and ultimate strain of the SMA fiber. While for the knotted end SMA fiber, increasing the bond length can improve the ultimate strain of the SMA fiber, but will reduce the maximum pullout stress. Increasing the fiber diameter can increase both maximum pullout stress and the ultimate strain of the SMA fiber with knotted ends.
4. SMA fibers with knotted ends placed in the ECC matrix show excellent self-centering ability, and the maximum self-centering ratio can reach 0.93. Increasing the bond length can result in a higher self-centering ratio but the difference is small. Additionally, the fiber diameter has no significant effect on the self-centering ability.

## Figures and Tables

**Table 1:** Material parameters of PVA fibers *.

Length /mm	Diameter /μm	Tensile Strength /MPa	Density /g/m^3^	Elastic Modulus /GPa	Elongation Ratio /%
9.0	31.0	1500.0	1.3	42.0	6.0

* Data from KURARAY Co., Ltd. (Shanghai, China).

**Table 2:** Mixture weight proportion of the ECC specimens.

Raw Materials	Cement	Fly Ash	Silica Sand	Water	PS	PVA */%
Mix proportion	1.0	2.4	0.36	0.26	0.0082	2.0

* Percentage of fibre content by volume.

**Table 3:** Uniaxial tensile mechanical properties of SMA wires.

Diameter/mm	Starting Point of Stress Platform		Ending Point of Stress Platform		Initial Elastic Modulus/MPa	Tensile Strength ^a^/MPa	Ultimate Strain ^b^/%
	Strain/%	Stress/MPa	Strain/%	Stress/MPa			
1.0	1.55	450.14	17.22	591.25	290.41	1123.10	28.63
1.2	1.65	468.65	13.74	581.26	284.03	1147.55	29.19
1.5	2.08	459.89	12.59	520.49	221.46	1372.77	25.42

^a^ The tensile strength is taken as the peak stress in the direct tensile stress–strain curve of SMA. ^b^ The ultimate strain is the maximum strain in the direct tensile stress–strain curve of SMA.

**Table 4:** Specimen grouping table.

Group	Specimen	Diameter/mm	End Shape	Bonding Length/mm
1	st-1.0	1.0	Straight	110 (through the matrix)
	st-1.2	1.2		
	st-1.5	1.5		
2	s-1.2-40	1.2	Straight	40
	h-1.2-40		Hooked	
	k-1.2-40		Knotted	
3	k-1.2-30	1.2	Knotted	30
	k-1.2-40			40
	k-1.2-50			50
4	k-1.0-40	1.0	Knotted	40
	k-1.2-40	1.2		
	k-1.5-40	1.5		

## Data Availability

Not applicable.
